# An evolutionary and functional assessment of regulatory network motifs

**DOI:** 10.1186/gb-2005-6-4-r35

**Published:** 2005-03-24

**Authors:** Aurélien Mazurie, Samuel Bottani, Massimo Vergassola

**Affiliations:** 1 Laboratoire de Génétique Moléculaire de la Neurotransmission et des Processus Neurodégénératifs CNRS UMR 7091, CERVI La Pitié, 91-105 boulevard de l'Hôpital, 75013 Paris, France; 2 Groupe de Modélisation Physique Interfaces Biologie and CNRS-UMR 7057 'Matières et Systèmes Complexes', Université Paris 7, 2 place Jussieu, 75251 Paris Cedex 05, France; 3 Unité Génomique des Microorganismes Pathogènes, CNRS URA 2171, Department of the Structure and Dynamics of Genomes, Institut Pasteur, 28 rue du Dr Roux, F-75724 Paris Cedex 15, France

## Abstract

**Background:**

Cellular functions are regulated by complex webs of interactions that might be schematically represented as networks. Two major examples are transcriptional regulatory networks, describing the interactions among transcription factors and their targets, and protein-protein interaction networks. Some patterns, dubbed motifs, have been found to be statistically over-represented when biological networks are compared to randomized versions thereof. Their function *in vitro *has been analyzed both experimentally and theoretically, but their functional role *in vivo*, that is, within the full network, and the resulting evolutionary pressures remain largely to be examined.

**Results:**

We investigated an integrated network of the yeast *Saccharomyces cerevisiae *comprising transcriptional and protein-protein interaction data. A comparative analysis was performed with respect to *Candida glabrata*, *Kluyveromyces lactis*, *Debaryomyces hansenii *and *Yarrowia lipolytica*, which belong to the same class of hemiascomycetes as *S. cerevisiae *but span a broad evolutionary range. Phylogenetic profiles of genes within different forms of the motifs show that they are not subject to any particular evolutionary pressure to preserve the corresponding interaction patterns. The functional role *in vivo *of the motifs was examined for those instances where enough biological information is available. In each case, the regulatory processes for the biological function under consideration were found to hinge on post-transcriptional regulatory mechanisms, rather than on the transcriptional regulation by network motifs.

**Conclusion:**

The overabundance of the network motifs does not have any immediate functional or evolutionary counterpart. A likely reason is that motifs within the networks are not isolated, that is, they strongly aggregate and have important edge and/or node sharing with the rest of the network.

## Abstract 2

Cross-species comparison and functional analysis of over-abundant motifs in an integrated network of yeast transcriptional and protein-protein interaction data showed that the over-abundance of the network motifs does not have any immediate functional or evolutive counterpart.

## Background

Global interaction data are synthetically structured as networks, their nodes representing the genes of an organism and their links some, usually indirect, form of interaction among them. This type of schematization is clearly wiping out important aspects of the detailed biological dynamics, such as localization in space and/or time, protein modifications and the formation of multimeric complexes, that have been lumped together in a link. Given these limitations, an important open question is whether the backbone of the interaction network provides any useful hints as to the organization of the web of cellular interactions. A first observation in this direction is that the topology of biological interaction networks strongly differs from that of random graphs [1]. In particular, when transcriptional regulatory networks are compared to randomized versions thereof, some special subgraphs, dubbed motifs, have been shown to be statistically over-represented [2,3]. An example of a motif composed of three units is the feed-forward loop, its name being inherited from neural networks, where this pattern is also abundant.

Transcription factors often act in multimeric complexes and the formation of these plays a crucial role in the regulatory dynamics. In order to capture at least part of those effects, transcriptional networks may be integrated with the protein-protein interaction data that have recently become available [4-7]. An example is provided by the mixed network constructed in [8]. The network is mixed in the sense that it includes both directed and undirected edges, pertaining to transcriptional and protein-protein interactions, respectively. The motifs for the mixed networks were investigated in [9].

The dynamics of motifs has been thoroughly investigated *in vitro *and *in silico*, that is, in the absence of the rest of the interaction network and of additional regulatory mechanisms [10-12]. For instance, the feed-forward loop has remarkable filtering properties, with the downstream-regulated gene activated only if the activation of the most-upstream regulator is sufficiently persistent in time. The motif essentially acts as a low-pass filter, with a time-scale comparable to the delay taken to produce the intermediate protein. Furthermore, the same structure is also found to help in rapidly deactivating genes once the upstream regulator is shut off. Overabundance of motifs and their interpretation as basic information-processing units popularized the hypothesis of an evolutionary selection of motifs [2,13].

In electrical engineering circuits, an abundant structure is likely to correspond to a module that performs a specific functional task and acts in a manner largely independent of the rest of the network. The point is moot for biological networks. A recent remark is that some of the motifs found in transcriptional networks are also encountered in artificial random networks [14,15], where no selection is acting. However, the lists of motifs do not entirely coincide for the two cases [16]. A visually striking fact is that essentially none of the motifs exists in isolation and that there is quite a great deal of edge-sharing with other patterns (see [17] for the network of *Escherichia coli*). The function of the motifs might then be strongly affected by their context. The use of genetic algorithms to explore the possible structures that perform a given functional task has in fact shown a wide variety of possible solutions [18].

It is therefore of interest to address the issue of the functional role of the motifs *in vivo*, that is within the whole network, and examine the ensuing evolutionary constraints. In the following, we shall show that the instances of the network motifs are not subject to any particular evolutionary pressure to be preserved and analyze the biological information available on the pathways where some instances of motifs are found.

## Results

### List and annotation of network motifs

**Figure 1:**
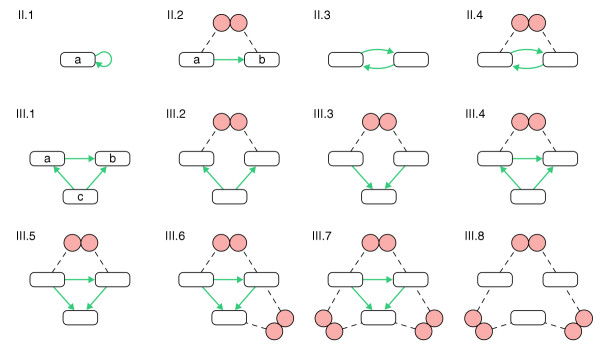
Types of motifs of order *n *= 2 and *n *= 3 for the mixed transcription and protein-protein network. The motifs shown here are those whose abundance patterns in the real network of the yeast *Saccharomyces cerevisiae *strongly deviate from the typical values found in randomized versions thereof. The green directed links with arrows represent transcriptional links, while two dashed lines with contacting circles represent an undirected protein-protein interaction.

The first step in the analysis of network motifs is their identification, as described in detail in Materials and methods. The patterns whose number of counts in the real network is found to significantly deviate from the typical values found in the randomized ensemble of the network are shown in Figure 1 (a generic representation of all the three-gene patterns independently of their statistical significance is given in Additional data file 1). The order of the patterns which we have examined are *n *= 2 and *n *= 3, where *n *is the number of genes of the pattern (see Materials and methods for the case of self-interactions).

The list includes the purely transcriptional feed-forward loop, investigated in [10-12], and its version augmented with a proteic interaction [9]. The overall list is quite similar to that found in [9], with the only exception of proteic self-interactions, which were not taken into account. General information on the motifs is obtained by looking at the biological processes, molecular functions and cellular components for which the genes found in occurrences of Figure 1 motifs have been annotated (see Additional data files 1 and 2).

Let us first remark that the various instances of the motifs account for 25% of all the genes annotated as transcription factors in the MIPS/FunCat and GeneOntology (GO) databases. The annotations obtained using the former database indicate that 34% of the genes involved in motifs are annotated as involved in transcriptional regulation and 31% in direct control of transcription; and that 51% of the genes have their products localized within the nucleus.

These values should be compared to 5% of all the genes annotated for transcriptional control in either GO or FunCat and 30% of nuclear localization for all annotated genes. Another relevant remark is that transcription factors are found at 93% and 11%, respectively, of the nodes with an outgoing and an ingoing transcriptional link. That is, indeed, the expected behavior for genes in a transcriptional network. These results witness the coherence of the transcription and the protein-protein interaction datasets used for finding the motifs and the published annotations.

As for the function of the genes composing the network motifs, the list of the most represented biological processes, as annotated in the MIPS database, is as follows: 50% of the genes are involved in metabolism, 34% in transcription, 21% in cell cycle and DNA processing, 12% in interaction with the cellular environment (10% in cellular sensing and response), 10% in cellular transport and 9% in rescue/defense.

**Figure 2:**
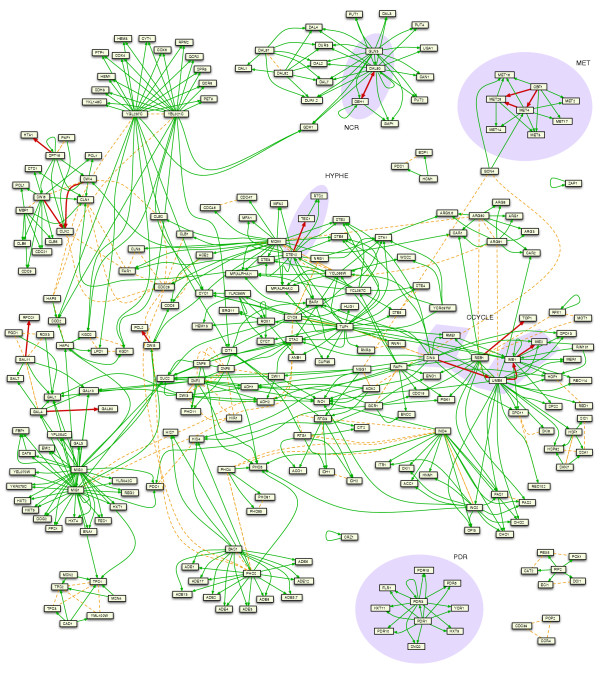
Motif occurrence in yeast. The network graph of the occurrences of motifs for *S. cerevisiae *illustrates the fact that most of the motifs are not found in isolation and are part of larger aggregates. Green, pure transcriptional regulation of the target gene by the regulatory gene product protein; red, transcriptional regulation and protein-protein interaction of the two partners; dashed line, pure protein-protein interaction. The pathways that will be examined in detail are shaded.

As shown clearly in Figure 2, motifs are generally combined into larger interaction sub-networks. Among the 504 instances of motifs in Figure 2, only four occur in isolation whereas all the others share genes and/or edges. This is also clear when we consider that only 256 different genes compose the 504 motif instances; 1,487 different genes would be possible if the instances were disjoint. Shared edges and/or genes and those forms of interactions not included in our database are likely to strongly affect the function of the motifs, raising the issue of their role *in vivo*. This will be the subject of the analysis presented in a further paper.

### Phylogenetic profiles of network motifs

To ascertain the presence of any special evolutionary pressure acting to preserve over-represented patterns, we have performed a protein comparative analysis between *Saccharomyces cerevisiae *and the four hemiascomycetes *Candida glabrata*, *Kluyveromyces lactis*, *Debaryomyces hansenii *and *Yarrowia lipolytica*, recently sequenced in [19]. The fact that the four organisms share many functional similarities with *S. cerevisiae *and yet span a broad range of evolutionary distances, comparable to the entire phylum of chordates, makes them ideal for protein comparisons. Details of the sequence comparisons are reported in Materials and methods.

Previous evolutionary studies on the motifs have explored the presence of common ancestors in different instances of the motifs. The upshot was that the various instances are not likely to have arisen by successive duplications of an ancestral pattern [20]. Here, we consider a different statistic based on the phylogenetic profiles [21] of the genes within the motifs. The profiles are constructed considering an ensemble of organisms and looking at the co-occurrences in the compared organisms of the genes composing the interaction pattern. This is quantified by the evolutionary fragility, *F*_*i *_(as defined in Materials and methods), of the interaction pattern *i*. A small value for the fragility indicates that the genes composing the pattern tend to co-occur in the other compared organisms, hinting at an evolutionary pressure to preserve the pattern and at its functional importance. We shall compare the statistics of the evolutionary fragility for different classes of interaction patterns, thus providing a test of the evolutionary significance of the criterion of overabundance used to identify network motifs.

**Figure 3:**
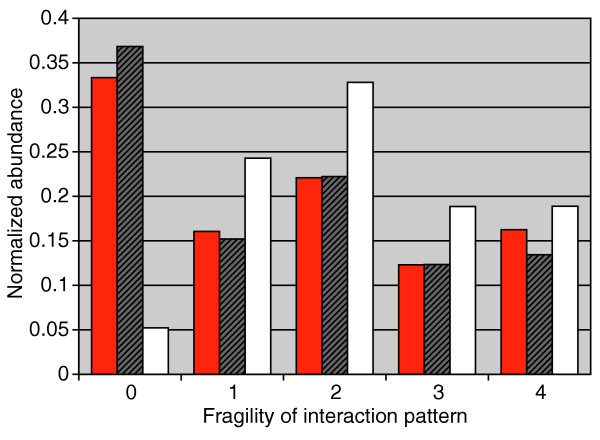
Phylogenetic profiles of interaction patterns. Normalized histograms of the evolutionary fragility of interaction patterns belonging to the following three classes are shown: instances of network motifs (red); generic patterns of interacting genes, irrespective of their abundance (black); patterns composed of genes taken at random (white). The five possible values (in increasing value 0 to 4) of the evolutionary fragility are reported on the abscissa. A small fragility value indicates that all the genes composing the interaction patterns tend to co-occur in the other genomes compared and point to evolutionary pressure acting to preserve the interaction pattern.

Specifically, in Figure 3 we report the normalized histograms of the evolutionary fragilities *F*_*i *_for three different classes of interaction patterns composed of three nodes: patterns which are instances of the motifs; all the interaction patterns, irrespective of their abundance; and patterns composed of genes taken at random. There are 481 instances of motifs in a total number of 9,962 patterns involving three nodes. Subtracting the 481 from the overall ensemble does not modify the conclusions drawn from Figure 3. The histogram for genes taken at random is clearly different from the other two, as expected. The point of interest to us here is that there is no statistically significant difference between the first two classes of patterns, as quantified by a *χ*^2 ^test, which gives *χ*^2 ^= 4.454 and a one-tailed probability 0.348. This clearly supports the hypothesis that the series of data for the two histograms are drawn from the same distribution. The conclusion of our comparative analysis is that instances of network motifs undergo no special evolutionary pressure as compared to a generic interaction pattern.

### Function *in vivo *of realizations of the motifs

**Figure 4:**
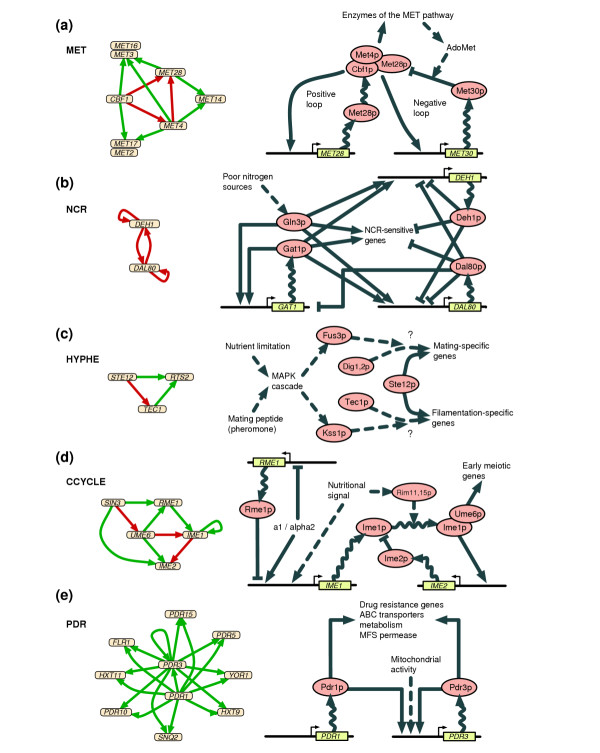
Outlines of the pathways studied. **(a) **Methionine (MET); **(b) **nitrogen catabolite repression (NCR); **(c) **pseudohypal growth/mating (HYPE); **(d) **regulation of early meiotic genes (CCYCLE); **(e) **pleiotropic drug resistance (PDR). The sub-networks enlarged from Figure 2, with the identified motifs within the pathway drawn from the interaction databases, are shown on the left (colors and conventions are the same as in Figure 2). A schematic representation of the regulation mechanisms for the same pathways, based on the present experimental knowledge as discussed in the text, is shown on the right. Full lines represent transcriptional regulation, dashed lines non-transcriptional regulation, and wavy lines transformations and syntheses. Arrowheads, positive regulation; lines ending in a terminal bar, negative regulation.

Biological information currently available is not sufficient to ascertain the function *in vivo *of all the occurrences of the motifs previously found. Some of them are, however, placed within well studied pathways and, in particular, a few of them are located at the interface between two blocks, one responsible for conveying a signal and the other for processing it. Two examples are the sub-networks methionine synthesis (MET) and nitrogen catabolite repression (NCR), shown shaded in Figure 2 and in more detail in Figure 4. The former, which is involved in methionine synthesis, receives a signal from the concentration of *S*-adenosylmethionine (AdoMet), a final metabolite of the sulfur amino acid pathway, and controls genes encoding enzymes involved in the pathway. The sub-network NCR, involved in nitrogen metabolism, receives a signal through the protein Gln3p, which is made available when nitrogen-rich sources are depleted, and controls genes encoding enzymes and transporters able to exploit alternative sources.

The importance of these pathways has made detailed biological information on their functions available. The interface location of the identified instances of the motifs raises the hope that they might be implicated in the dynamics of the information processing and, in particular, that the time-filter properties mentioned above might be exploited to control the time-response processing of the external signal. Ascertaining this behavior was our motive for investigating the detailed functioning of each of the pathways. We report here the principles of the core regulatory mechanisms involved in the chosen pathways, referring the reader to the cited literature for a detailed treatment. Here we are interested in identifying the possible role of motifs in biological functions.

### The methionine pathway

Sub-network MET in Figures 2 and 4a shows the interaction graph for the cluster of interacting genes centered on *CBF1*, *MET4 *and *MET28*. The graph includes three motifs of type II.2, five of type III.5 and one of type III.7 (see Figure 1 for motif types). The methionine biosynthesis network has been thoroughly investigated [22-25] and a detailed biological model of the pathway is now available. Cbf1p, Met4p and Met28p form a heterotrimer that activates target genes of the sulfur pathway (MET genes). Inside the complex, only Met4p has direct transcriptional action, with Cbf1p being involved in chromatin rearrangement and Met28p tethering the complex to the DNA. The MET genes are activated by the complex, but are repressed when one of the final metabolites of the pathway, AdoMet, increases. Two loops drive the dynamics of complex availability, sketched in Figure 4a. One is a positive loop: the Met4p complex regulates the transcription of *MET28*, its product stimulating the tethering of the complex to DNA. This loop is responsible for the increase of the dynamic response when the intracellular AdoMet concentration is low (the transcription of *MET4 *is constitutive). The other is a negative loop: Met4p controls its own fate by regulating the transcription of *MET30*. The product of the latter is an ubiquitin ligase, which triggers the degradation of Met4p when AdoMet increases. This loop is expected to control high detrimental accumulation of AdoMet.

Note that the latter post-transcriptional mechanism is, by definition, not captured by the network, which is limited to transcriptional regulations. Furthermore, an intrinsic limitation of network structures should be noted: the three proteins Cbf1p, Met4p and Met28p always act as a complex. This information does not unambiguously emerge from the topology of the network (Figure 4a, left), as the topology is also compatible with the three proteins acting separately. In conclusion, the key features of the methionine synthesis pathway do not seem to hinge on transcriptional regulation via the motifs instances shown in Figure 4a.

### Nitrogen catabolite repression (NCR) system

The NCR system shown in Figures 2 and 4b is used by the cell to control the synthesis of proteins capable of handling poor sources of nitrogen. NCR-sensitive genes are not activated when rich sources are available, whereas they get expressed when only poor sources are left. Two II.1 and one II.4 motifs are embedded in this system.

*DEH1 *and *DAL80 *are part of the GATA gene family and are known transcriptional repressors, regulating nitrogen catabolite repression via their binding to the GATA sequences upstream of NCR-sensitive genes. For several targets, the two repressors are in competition with Gln3p and Gat1p, which are transcriptional activators binding the same sequences.

The accepted mechanisms of NCR are as follows ([26-28] and see Figure 4b). First, in the presence of rich nitrogen sources (ammonia and/or glutamine), Gln3p and Gat1p are sequestered in the cytoplasm and can activate neither NCR-sensitive genes nor *DEH1 *and *DAL80*. The consequence of the low concentration of Gln3p in the nucleus is a low-level expression of *DEH1*, *DAL80 *and NCR-sensitive genes. Second, when poor sources only are available (such as urea, prolin, or GABA), Gln3p and Gat1p are released into the nucleus. The former activates *GAT1 *and the two proteins together activate NCR-sensitive genes. After a delay (due to the time taken for transcription and translation), Dal80p and Deh1p are expressed and competitively inhibit these same genes.

Interesting dynamic behavior takes place during a transition from rich to poor nitrogen sources, when the cell must cast about for alternative sources, which implies the synthesis of new proteins. The amount of these proteins synthesized must be sufficient to ensure utilization of the new sources but, because of the depletion of nutrient sources, they should not be too high. NCR-sensitive genes are therefore activated only for the limited period of time when Gln3p and Gat1p are present but Dal80p and Deh1p are not. The negative feedback of *DAL80 *on its activator *GAT1 *is the mechanism ensuring that oscillatory behavior.

To summarize, the role of the motifs identified in the NCR system is not evident and the entire mechanism of the NCR, within the model currently accepted on the basis of the present knowledge, can be described without any reference to them.

### Pseudohyphal growth/mating MAPK system

The sub-network HYPHE in Figure 2 and Figure 4c is formed by one motif of type III.5, involving the two genes *STE12 *and *TEC1*. These genes both code for a transcription factor and are located downstream of the mitogen-activated protein kinase (MAPK) signal transduction pathway that controls both the pseudohyphal growth of the yeast and its mating response to pheromones. These signal transductions constitute a striking example of a signaling pathway shared by two different signals and yet responding specifically to each of them. It is therefore the object of detailed investigation and much data are available [29]. The phenomenology of the regulatory process is summarized as follows: in response to pheromones, Ste12p binds specifically to the pheromone response elements (PRE) of genes involved in the mating process; under conditions of starvation, a heterodimer composed of Tec1p and Ste12p binds to genes involved in pseudohyphal growth.

The fact that *STE12 *regulates *TEC1 *raises the possibility that the switch between the two shared pathways of response to pheromones and pseudohyphal growth be realized by the instance of the feed-forward III.5 motif in the HYPHE sub-network. However, there is quite clear evidence that this is not the case, the most direct indication being provided in [30], where it is shown that the level of expression of *TEC1 *does not correlate with pseudohyphal growth. Recent work indicates that the switch is instead realized via post-transcriptional phosphorylation effects, controlled by the two kinases Fus3p and Kss1p, and affecting the multimerization of Ste12p. Fus3p and Kss1p constitute the final layer of the MAPK system and are differentially activated in the two pathways (see, for example [31]).

### Regulation of early meiotic genes

The sub-network around *IME1 *in Figure 2 and Figure 4d is made of one II.1, two III.5 and one III.6 motifs and is implicated in the activation of early meiotic genes. The process of regulation of entry into meiosis and the early activation of the relevant genes has been studied in great detail and is summarized in [32]. In short, the meiotic pathway in yeast is initiated by the expression and activation of *IME1*, which serves as the master regulatory switch for meiosis [33]. Expression of *IME1 *requires the integration of a genetic signal, indicating that the cell is diploid, and a nutritional signal, indicating that the cell is starved. The point of interest here is to ascertain if the processing of these signals takes place at the transcriptional level by the instances of the motifs in the sub-network. This does not seem to be the case. The information processing is rather implemented by alternative routes and the picture of the interactions shown on the sub-network CCYCLE in Figure 2 and Figure 4d (left) appears to be insufficient and misleading.

The repression of *IME1 *by *RME1 *has a major role in cell-type control, and *IME1 *expression does not involve the regulation of *RME1 *by the complex Ume6p-Sin3p, as suggested by the sub-network CCYCLE in Figure 2. This is realized through the cell-type specific a1 and *α *2 proteins, which combine in diploid cells and bind specifically to sites in the promoter of *RME1 *to repress its expression [32,33].

The integration of the nutritional signal is processed by both *IME1 *and *IME2 *and is considerably more complex than cell-type regulation, its main steps being reviewed in [34]. For instance, the *IME1 *promoter has at least 10 separate regulatory elements. *IME2 *is also regulated by several distinct signals, integrated at a single regulatory element, the upstream repression site *URS1*, which is bound by the Ume6p transcription factor under all conditions tested. The activation of *IME1 *and *IME2 *depends on the multimerization of Ume6p with several other proteins regulated either positively or negatively by at least two kinases, Rim11p and Rim15p. Other non-transcriptional mechanisms of gene control (such as targeted degradation) appear also to be involved in the regulation of this process [35]. The motifs in the sub-network CCYCLE fail to capture the complexity of these interwoven interactions.

### Pleiotropic drug resistance (PDR) system

The PDR system is used by the cell to counter the action of a broad spectrum of toxic substances; by activating membrane efflux pumps and modifying the membrane composition, the concentration of these substances is then decreased. Two genes, *PDR1 *and *PDR3*, encode homologous transcription factors [36,37], which drive multidrug resistance by activating genes involved in active transport and lipid metabolism [38,39].

The corresponding sub-network (named PDR in Figure 2 and 4e) is composed of eight motifs of type III.1 (so-called feed-forward loops) and one of type II.1, showing a star-like configuration with *PDR1 *and *PDR3 *in a central position.

*In vivo*, those two genes have apparent functional redundancy: they target the same genes and the deletion of either *PDR1 *or *PDR3 *does not significantly affect the PDR system; an effect is only shown when both are deleted [40,41]. However, these two factors are used in response of two different cell signals: *PDR3 *is sensitive to mitochondrial activity, whereas *PDR1 *is not [42-44]. Conversely, *PDR1 *deletion mutants are quite drug-hypersensitive, whereas *PDR3 *mutants are not [41].

In addition to this distinct response of *PDR1 *and *PDR3 *to cellular signals, the regulation link between them is weak, and no proof of cooperativity for the regulation of their targets was highlighted.

It the PDR sub-network, the III.1 motifs formed by *PDR1*, *PDR3 *and their common targets are apparently not exploited by the cell because *PDR1 *and *PDR3 *are not obligatorily active at the same time and the prerequisites for the specific dynamics of feed-forward loops are not fulfilled (sufficient regulation of *PDR3 *by *PDR1 *and cooperativity on the common targets).

## Discussion

The motivating idea behind most discussions on motifs is the possibility of capturing the essential logic of genetic regulation by a small set of interaction circuits performing some specific functional tasks. While this hypothesis is, in principle, experimentally testable, experimental and theoretical work has hitherto considered essentially motifs in isolation, that is, excised from the biological environment in which the motifs' instances are embedded.

We studied in detail the role of motifs in the case of the best-documented genetic sub-networks and biological functions where such motifs are found. In most cases, motifs do not seem to have a central regulatory role in the biological processes associated with each occurrence. The list of examples where enough biological information is available is, of course, limited, and further examples may subvert this picture. At the moment, it is a fact that all the examples studied highlight the high level of integration of different regulatory mechanisms acting altogether. Reception and processing of cellular signals cannot be reduced to transcriptional regulation and protein-protein interaction switches. Other mechanisms such as phosphorylation, triggered degradation, protein sequestration and transport, and higher-order multimerization are central to the logic of the sub-networks. Disentangling information-processing circuits made of transcription reactions and interactions between transcription factors from the whole cellular environment does not seem to be possible for the cases considered. A qualitative impression surmised from the visible aggregation and nesting of the motifs with the rest of the network is that a 'pure' modular functional behavior is not very likely to occur. This impression is not limited to *S. cerevisiae*: in previous work [17], other researchers have shown that a similar aggregation of structural motifs occurs for a simpler organism, *E. coli*, suggesting some degree of generality.

Some comments on structuring interaction data in the form of topological networks are worth making. The graph is indeed an abstraction constructed from available databases and its meaning is influenced by several factors. For instance, the graph is a static projection of possible interactions. The analysis of regulatory processes varying in space and time requires additional information not usually included in the topology of biological networks. Indeed, the very representation in the form of a unique network entails the integration in space and time of the interactions taking place during the cellular lifetime. Some of the patterns of interaction might then be spuriously due to a projection effect, whereas they actually take place at different times and/or locations within the cell. This is occurring, for example, in the PDR system: *PDR1 *and *PDR3 *at the base of the eight III.1 motifs respond to different signals and control their outputs independently (no cooperation on the common targets). These motifs appear in the network because different conditions at different times were projected onto the same plane.

Furthermore, the patterns in the network may be a direct consequence of the data models in the current databases, and incorrectly represent the biological context. Transitory macromolecular associations like protein complexes and interactions between a whole protein complex and a target are indeed missed, and at most represented as individual links between each component and the target. This is what occurs with the Met4p/Met28p/Cbf1p heterotrimer, which appears in the network as three independent interacting components together with three III.5 motifs that do not actually exist.

The NCR system is an interesting example where motifs are clearly identified and seem unambiguous. However, to the best of our knowledge they do not play any significant role. In particular, the role of the mutual interactions between *DAL80 *and *DEH1 *(sustaining a II.4 motif) is not clear. An intriguing hypothesis is that the presence of the interactions might be traced back to the strong sequence similarity between *DAL80 *and *DEH1*. The products of both these genes form homodimers and inhibit their own expression. The presence of the motif might then be due to a recent duplication event, which has therefore preserved the interactions.

Divergent evolution seems also to be the origin of the appearance of motifs in the PDR system. In this case, the two diverging genes *PDR1 *and *PDR3 *have acquired different independent functions. The motif instance that they form together is the apparently unexploited consequence of their common origin.

## Conclusion

The results presented here indicate that the statistical abundance of network motifs has no evident counterpart at the evolutionary and *in vivo *functional level. Occurrences of network motifs have indeed been shown to possess the same evolutionary fragility; that is, when different organisms are compared, the genes composing the motif have similar co-occurrence profiles as genes in interaction patterns with a normal abundance.

The point seems to be confirmed by the analysis of the functional role of examples of the motifs occurrences. These are located at the interface between two blocks - one responsible for the reception of a signal and the other for its processing - and have been selected because detailed biological information on those pathways is available. The number of cases is limited, but in none of them are the major steps of signal information processing taking place at the transcriptional level through the implementation of the motifs. Alternative routes involving post-transcriptional regulation and intracellular compartmentalization seem to be exploited for this purpose.

These results naturally bring up the issue as to the actual role of the motifs. Some occurrences have been shown to arise spuriously from the representation of the interaction data in the form of a network and the ensuing projection effects in space and/or time. It seems, however, fair to assume that those effects should be limited to a few cases. The metabolic costs of producing proteins and the fact that some of the motifs instances examined are active in conditions of starvation make it likely that proteins encoded by genes composing these motifs do play a role. What is however quite clear from Figure 2 and our analysis is that the great majority of motif occurrences are in fact embedded in larger structures and entangled with the rest of the network. Only a small minority is isolated and likely to perform a specific functional task that does not depend on the context.

This clustering is important as it indicates that the choice of the null model used to gauge the statistical importance of the abundance of interaction patterns might be delicate. Indeed, the higher-order context is not taken into account in the randomization process used to generate the null model networks, and we have shown that this is manifestly not a choice ensuring a strong evolutionary and (*in vivo*) functional significance. Accounting for the various layers of organization of biological networks seems crucial to correctly identify the functional elements responsible for the information processing that allows living cells to cope with their highly variable environmental conditions.

## Materials and methods

### Datasets

The transcriptional regulatory network used for the analysis is the one constructed and investigated in [45]. It was preferred to the more extended one derived from ChIP-chips data in [46] as the fraction of links where the regulatory role of the various interactions is documented is higher for the former. The protein-protein interaction data in the Database of Interacting Proteins (DIP [47]) are a large collection of both two-hybrid and TAP-tag data. The resulting network has 476 nodes, 905 directed transcriptional edges and 221 undirected protein-protein edges.

### Identification of motifs and network randomization

The detection of *n*-node network motifs is performed along lines similar to those used in [2]. The method exhaustively scans the neighborhood of all the links in the network to search for the motif of interest, and then purges the list for repeated patterns.

Randomized versions of the network are generated as follows. Links are swapped as in the Markov-chain algorithm used in [48], that is, two links between the couples of nodes (*X*_1_*Y*_1_) and (*X*_2_*Y*_2_) are replaced by (*X*_1_*Y*_2_) and (*X*_2_*Y*_1_). In our case, where the links might be transcriptional or protein-protein interaction, the links that are swapped must be of the same type. This procedure is guaranteed to preserve the single-point connectivity at each node of the network.

As for the randomization procedure for *n *= 3 motifs, we want to avoid the possibility that higher-order motifs spuriously inherit statistical significance from lower orders. In other words, the randomized network ought to have the same statistics for all the patterns of order *n *= 2 as the real network. This is ensured by converging a simulated annealing, where the elementary steps are the swappings of the links previously described. The transition probabilities are weighted according to the difference:



where the sum runs over all the patterns of order *n *= 2 and the *c*_*i *_values denote the number of patterns in the two types of networks.

Statistically significant patterns are those where the number of counts has a low probability to be observed in the ensemble of networks obtained by randomization. Specifically, we require that the observed number of counts , has a one-tailed probability:



- or the opposite inequality if the pattern is under-represented in the real network - to occur in the randomized ensemble. The probabilities are estimated from a Monte-Carlo sampling of 10,000 trials of the randomized ensemble distribution and the results are sensitive neither to the number of trials nor to the thresholds chosen. The probability distribution functions are often found to deviate from a Gaussian curve and the one-tailed probabilities are therefore directly measured from the normalized histograms without relying on z-scores.

Note that patterns involving self-interactions are somewhat special, as their order *n*, which controls the type of random networks they should be compared to, does not coincide with their number of genes. For example, a single gene self-interacting is treated as an *n *= 2 pattern. The reason is that a sensible way of assessing the significance for this pattern is by having a fixed number of total proteic links and studying the fraction of them that are self-interactions. In other words, self-interactions are swapped throughout the randomization procedure with proteic links between two distinct proteins and their order is therefore *n *= 2.

### Sequence comparisons

BLAST searches were performed using BLASTP 2.2.6 [49] with the BLOSUM 62 matrix and affine gap penalties of 11 (gap) and 1 (extension). Putative orthologs were inferred from the primary sequence and keeping only bidirectional best hits to reduce the effect of the high number of paralogs in yeast genomes. Tables of bidirectional best hits were constructed by identifying the pairs of proteins in the two organisms compared which are the reciprocal best alignments. The significance of the alignments was quantified by the BLAST e-values and different thresholds were considered, ranging from 10^-1 ^to 10^-10^. Their choice does not affect the results presented in the body of the paper.

### Evolutionary fragility of interaction patterns

Let us consider all the interaction patterns, indexed by *i*, composed of interacting genes of *S. cerevisiae *and each one of the other four hemiascomycetes, indexed by *α*. The boolean variable *f*_*iα *_for the pattern *i *is taken equal to zero if the genes composing the pattern are all present/absent in the other organism *α *and is unity otherwise. Presence/absence is measured by using the list of bidirectional best hits discussed in the previous section. The selective pressure to preserve the pattern *i *is quantified by the fragility:



The two extreme cases are *F*_*i *_= 0 and *F*_*i *_= 4 (the number of organisms compared). The two cases correspond to the genes composing the pattern co-occurring in all or none of the compared organisms, respectively. As an additional example, consider the case where the three genes composing an interaction pattern are all present in *C. glabrata*, *K. lactis *and *D. hansenii *(which are evolutionarily closer to *S. cerevisiae*) but one (or two) of them is absent in *Y. lipolytica*. The corresponding value of the fragility is *F*_*i *_= 1.

## Additional data files

Additional data are available with the online version of this paper. Additional data file 1 is a figure showing general three-gene patterns. Additional data file 2 is a table showing motif occurrences. Additional data file 3 is a table showing functions of the genes in motif occurrences.

## Supplementary Material

Additional File 1 Left: the two possible connectivity topologies between three genes. Each grey line can be any of the seven types of interaction represented on the right. Right: the different types of interaction between two genes and their products. Boxes: genes; green arrows: transcriptional regulation only; dashed lines with circles: protein-protein interaction of the genes products. 1-3: only transcriptional regulation without known ppi interaction, (1 and 2 are distinguished to account for different combinations in the diagrams on the left). 4: protein-protein interaction only. 5-7: transcriptional regulation and interaction between the genes products (without details on the role of the ppi interaction complex). The set of all possible three genes patterns is obtained with all the combinations of the interaction types shown on the right on the topologies shown on the left. The statistically significant patterns form a subset of 8 three genes motifs shown in Figure 1 Click here for file

Additional File 2 The list of the motif instances found for the yeast Saccharomyces cerevisiae. Each line corresponds to a different realization and contains the most used non-ambiguous name of the involved genes, ordered according to their position in the motif. First column contains the motifs type according to Figure 1; columns 2 to 4 correspond respectively to the genes positions a, b and c as indicated in figure 1 Click here for file

Additional File 3 The Excel file contains the list of genes found in motif realizations with their biological functions as given by the MIPS database using the FunCat ontology, and different statistics on function occurrences and distribution. The data is presented in three sheets with different viewpoints: **First sheet, "Functions by genes": **gives a list of all genes found in motifs instances with standard, main and alternative names, motifs and positions within motifs where these genes are found (according to types and positions as defined in Figure 1), and finally biological functions. **Second sheet, "Functions by positions": **gives motifs and positions within motifs grouped according to functions. For each represented function in FunCat, the first three columns indicate the number, the fraction and the names of the genes found in motifs instances having this function. The following columns indicate the details for each motif type with: the number of genes involved in the given motif with the given function, the fraction of all genes within motifs having this position and this function, the fraction of genes for this function that are at this position, and the fraction of genes at this position having this function. **Third sheet, "Genes by positions": **gives standard and main name of genes found at each position Click here for file

## References

[B1] Jeong H, Mason S, Barabási A, Oltvai Z (2001). Lethality and centrality in protein networks.. Nature.

[B2] Milo R, Shen-Orr S, Itzkovitz S, Kashtan N, Chklovskii D, Alon U (2002). Network motifs: simple building blocks of complex networks.. Science.

[B3] Shen-Orr SS, Milo R, Mangan S, Alon U (2002). Network motifs in the transcriptional regulation network of *Escherichia coli*.. Nat Genet.

[B4] Uetz P, Giot L, Cagney G, Mansfield T, Judson R, Knight J, Lockshon D, Narayan V, Srinivasan M, Pochart P (2000). A comprehensive analysis of protein-protein interactions in *Saccharomyces cerevisiae*.. Nature.

[B5] Ito T, Chiba T, Ozawa R, Yoshida M, Hattori M, Sakaki Y (2001). A comprehensive two-hybrid analysis to explore the yeast protein interactome.. Proc Natl Acad Sci USA.

[B6] Gavin AC, Bösche M, Krause R, Grandi P, Marzioch M, Bauer A, Schultz J, Rick JM, Michon AM, Cruciat CM (2002). Functional organization of the yeast proteome by systematic analysis of protein complexes.. Nature.

[B7] Ho Y, Gruhler A, Heilbut A, Bader GD, Moore L, Adams SL, Millar A, Taylor P, Bennett K, Boutilier K (2002). Systematic identification of protein complexes in Saccharomyces cerevisiae by mass spectrometry.. Nature.

[B8] Yeger-Lotem E, Margalit H (2003). Detection of regulatory circuits by integrating the cellular networks of protein-protein interactions and transcription regulation.. Nucleic Acids Res.

[B9] Yeger-Lotem E, Sattath S, Kashtan N, Itzkovitz S, Milo R, Pinter RY, Alon U, Margalit H (2004). Network motifs in integrated cellular networks of transcription-regulation and protein-protein interaction.. Proc Natl Acad Sci USA.

[B10] Rosenfeld N, Elowitz MB, Alon U (2002). Negative autoregulation speeds the response times of transcription networks.. J Mol Biol.

[B11] Mangan S, Alon U (2003). Structure and function of the feed-forward loop network motif.. Proc Natl Acad Sci USA.

[B12] Mangan S, Zaslaver A, Alon U (2003). The coherent feedforward loop serves as a sign-sensitive delay element in transcription networks.. J Mol Biol.

[B13] Milo R, Itzkovitz S, Kashtan N, Levitt R, Shen-Orr S, Ayzenshtat I, Sheffer M, Alon U (2004). Superfamilies of evolved and designed networks.. Science.

[B14] Artzy-Randrup Y, Fleishman SJ, Ben-Tal N, Stone L (2004). Comment on "Network motifs: simple building blocks of complex networks" and "Superfamilies of evolved and designed networks".. Science.

[B15] Banzhaf W, Kuo PD (2004). Network motifs in natural and artificial transcriptional regulatory networks.. J Biol Phys Chem.

[B16] Milo R, Itzkovitz S, Kashtan N, Levitt R, Alon U (2004). Response to Comment on "Network Motifs: Simple Building Blocks of Complex Networks" and "Superfamilies of Evolved and Designed Networks".. Science.

[B17] Dobrin R, Beg QK, Barabási AL, Oltvai ZN (2004). Aggregation of topological motifs in the *Escherichia coli *transcriptional regulatory network.. BMC Bioinformatics.

[B18] François P, Hakim V (2004). Design of genetic networks with specified functions by evolution *in silico*.. Proc Natl Acad Sci USA.

[B19] Dujon B, Sherman D, Fischer G, Durrens P, Casaregola S, Lafontaine I, Montigny JD, Marck C, Neuvéglise C, Talla E (2004). Genome evolution in yeasts.. Nature.

[B20] Conant GC, Wagner A (2003). Convergent evolution of gene circuits.. Nat Genet.

[B21] Pellegrini M, Marcotte E, Thompson M, Eisenberg D, Yeates T (1999). Assigning protein functions by comparative genome analysis: protein phylogenetic profiles.. Proc Natl Acad Sci USA.

[B22] Thomas D, Surdin-Kerjan Y (1997). Metabolism of sulfur amino acids in *Saccharomyces cerevisiae*.. Microbiol Mol Biol Rev.

[B23] Kuras L, Barbey R, Thomas D (1997). Assembly of a bZIP-bHLH transcription activation complex: formation of the yeast Cbf1-Met4-Met28 complex is regulated through Met28 stimulation of Cbf1 DNA binding.. EMBO J.

[B24] Blaiseau P, Thomas D (1998). Multiple transcriptional activation complexes tether the yeast activator Met4 to DNA.. EMBO J.

[B25] Rouillon A, Barbey R, Patton E, Tyers M, Thomas D (2000). Feedback-regulated degradation of the transcriptional activator Met4 is triggered by the SCF(Met30) complex.. EMBO J.

[B26] Cooper TG (2002). Transmitting the signal of excess nitrogen in *Saccharomyces cerevisiae *from the Tor proteins to the GATA factors: connecting the dots.. FEMS Microbiol Rev.

[B27] Cox KH, Tate JJ, Cooper TG (2002). Cytoplasmic compartmentation of Gln3 during nitrogen catabolite repression and the mechanism of its nuclear localization during carbon starvation in *Saccharomyces cerevisiae*.. J Biol Chem.

[B28] Cunningham T, Rai R, Cooper T (2000). The level of DAL80 expression down-regulates GATA factor-mediated transcription in *Saccharomyces cerevisiae*.. J Bacteriol.

[B29] Barolo S, Posakony JW (2002). Three habits of highly effective signaling pathways: principles of transcriptional control by developmental cell signaling.. Genes Dev.

[B30] Oehlen L, Cross F (1998). The mating factor response pathway regulates transcription of TEC1, a gene involved in pseudohyphal differentiation of *Saccharomyces cerevisiae*.. FEBS Lett.

[B31] Zeitlinger J, Simon I, Harbison CT, Hannett NM, Volkert TL, Fink GR, Young RA (2003). Program-specific distribution of a transcription factor dependent on partner transcription factor and MAPK signaling.. Cell.

[B32] Pringle J, Broach J, Jones E, (Eds) (1997). The Molecular and Cellular Biology of the Yeast Saccharomyces Cell Cycle and Cell Biology.

[B33] Vershon A, Pierce M (2000). Transcriptional regulation of meiosis in yeast.. Curr Opin Cell Biol.

[B34] Honigberg SM, Purnapatre K (2003). Signal pathway integration in the switch from the mitotic cell cycle to meiosis in yeast.. J Cell Sci.

[B35] Guttmann-Raviv N, Martin S, Kassir Y (2002). Ime2, a meiosis-specific kinase in yeast, is required for destabilization of its transcriptional activator, Ime1.. Mol Cell Biol.

[B36] Balzi E, Chen W, Ulaszewski S, Capieaux E, Goffeau A (1987). The multidrug resistance gene PDR1 from *Saccharomyces cerevisiae*.. J Biol Chem.

[B37] Delaveau T, Jacq C, Perea J (1992). Sequence of a 12.7 kb segment of yeast chromosome II identifies a PDR-like gene and several new open reading frames.. Yeast.

[B38] DeRisi J, vanden Hazel B, Marc P, Balzi E, Brown P, Jacq C, Goffeau A (2000). Genome microarray analysis of transcriptional activation in multidrug resistance yeast mutants.. FEBS Lett.

[B39] Devaux F, Marc P, Bouchoux C, Delaveau T, Hikkel I, Potier M, Jacq C (2001). An artificial transcription activator mimics the genome-wide properties of the yeast Pdr1 transcription factor.. EMBO Rep.

[B40] Delaveau T, Delahodde A, Carvajal E, Subik J, Jacq C (1994). PDR3, a new yeast regulatory gene, is homologous to PDR1 and controls the multidrug resistance phenomenon.. Mol Gen Genet.

[B41] Katzmann D, Burnett P, Golin J, Mahé Y, Moye-Rowley W (1994). Transcriptional control of the yeast PDR5 gene by the PDR3 gene product.. Mol Cell Biol.

[B42] Hallstrom T, Moye-Rowley W (2000). Multiple signals from dysfunctional mitochondria activate the pleiotropic drug resistance pathway in *Saccharomyces cerevisiae*.. J Biol Chem.

[B43] Zhang X, Moye-Rowley W (2001). *Saccharomyces cerevisiae *multidrug resistance gene expression inversely correlates with the status of the F(0) component of the mitochondrial ATPase.. J Biol Chem.

[B44] Devaux F, Carvajal E, Moye-Rowley S, Jacq C (2002). Genome-wide studies on the nuclear PDR3-controlled response to mitochondrial dysfunction in yeast.. FEBS Lett.

[B45] Guelzim N, Bottani S, Bourgine P, Képès F (2002). Topological and causal structure of the yeast transcriptional regulatory network.. Nat Genet.

[B46] Lee TI, Rinaldi NJ, Robert F, Odom DT, Bar-Joseph Z, Gerber GK, Hannett NM, Harbison CT, Thompson CM, Simon I (2002). Transcriptional regulatory networks in *Saccharomyces cerevisiae*.. Science.

[B47] Database of Interacting Proteins. http://dip.doe-mbi.ucla.edu.

[B48] Maslov S, Sneppen K (2002). Specificity and stability in topology of protein networks.. Science.

[B49] Altschul S, Madden T, Schäffer A, Zhang J, Zhang Z, Miller W, Lipman D (1997). Gapped BLAST and PSI-BLAST: a new generation of protein database search programs.. Nucleic Acids Res.

